# Reducing readmission rates for individuals discharged from acute psychiatric care in Alberta using peer and text message support: Protocol for an innovative supportive program

**DOI:** 10.1186/s12913-022-07510-8

**Published:** 2022-03-12

**Authors:** Ejemai Eboreime, Reham Shalaby, Wanying Mao, Ernest Owusu, Wesley Vuong, Shireen Surood, Kerry Bales, Frank P. MacMaster, Diane McNeil, Katherine Rittenbach, Arto Ohinmaa, Suzette Bremault-Phillips, Carla Hilario, Russ Greiner, Michelle Knox, Janet Chafe, Jeff Coulombe, Li Xin-Min, Carla McLean, Rebecca Rathwell, Mark Snaterse, Pamela Spurvey, Valerie H Taylor, Susan McLean, Liana Urichuk, Berhe Tzeggai, Christopher McCabe, David Grauwiler, Sara Jordan, Ed Brown, Lindy Fors, Tyla Savard, Mara Grunau, Frank Kelton, Sheila Stauffer, Bo Cao, Pierre Chue, Adam Abba-Aji, Peter Silverstone, Izu Nwachukwu, Andrew Greenshaw, Vincent Israel Opoku Agyapong

**Affiliations:** 1 Department of Psychiatry, Faculty of Medicine and Dentistry, University of Alberta, Edmonton, AB Canada; 2 Alberta Health Services, Addiction and Mental Health Services, Edmonton Zone, AB Canada; 3 Alberta Health Services, Provincial Addiction & Mental Health Portfolio, Edmonton, AB Canada; 4 Department of Psychiatry, Cumming School of Medicine, University of Calgary, Calgary, AB Canada; 5 School of Public Health, University of Alberta, Edmonton, AB Canada; 6 Heroes in Mind, Advocacy and Research Consortium (HiMARC), Faculty of Rehabilitation Medicine, University of Alberta, Edmonton, AB Canada; 7 Faculty of Nursing, University of Alberta, Edmonton, AB Canada; 8 Department of Computing Science, Faculty of Science, University of Alberta, Edmonton, AB Canada; 9 Institute for Health Economics, Edmonton, AB Canada; 10 Canadian Mental Health Association, Edmonton, AB Canada; 11 Patients’ Advisory Council, Addiction and Mental Health, Alberta Health Services, Edmonton, AB Canada; 12 Centre for Suicide Prevention, Calgary, AB Canada; 13 Potential Place Society, Calgary, AB Canada; 14 Cornerstone Counselling, Edmonton, AB Canada; 15 Department of Psychiatry, Faculty of Medicine, Dalhousie University, 5909 Veterans Memorial Lane, 8th Floor, Abbie J. Lane Memorial Building, QEII Health Sciences Centre, Halifax, NS B3H 2E2 Canada

**Keywords:** Mental health, Hospital readmission, Text4Support, Peer support

## Abstract

**Background:**

Individuals discharged from inpatient psychiatry units have the highest readmission rates of all hospitalized patients. These readmissions are often due to unmet need for mental health care compounded by limited human resources. Reducing the need for hospital admissions by providing alternative effective care will mitigate the strain on the healthcare system and for people with mental illnesses and their relatives. We propose implementation and evaluation of an innovative program which augments Mental Health Peer Support with an evidence-based supportive text messaging program developed using the principles of cognitive behavioral therapy.

**Methods:**

A pragmatic stepped-wedge cluster-randomized trial, where daily supportive text messages (Text4Support) and mental health peer support are the interventions, will be employed. We anticipate recruiting 10,000 participants at the point of their discharge from 9 acute care psychiatry sites and day hospitals across four cities in Alberta. The primary outcome measure will be the number of psychiatric readmissions within 30 days of discharge. We will also evaluate implementation outcomes such as reach, acceptability, fidelity, and sustainability. Our study will be guided by the Consolidated Framework for Implementation Research, and the Reach-Effectiveness-Adoption-Implementation-Maintenance framework. Data will be extracted from administrative data, surveys, and qualitative methods. Quantitative data will be analysed using machine learning. Qualitative interviews will be transcribed and analyzed thematically using both inductive and deductive approaches.

**Conclusions:**

To our knowledge, this will be the first large-scale clinical trial to assess the impact of a daily supportive text message program with and without mental health peer support for individuals discharged from acute psychiatric care. We anticipate that the interventions will generate significant cost-savings by reducing readmissions, while improving access to quality community mental healthcare and reducing demand for acute care. It is envisaged that the results will shed light on the effectiveness, as well as contextual barriers and facilitators to implementation of automated supportive text message and mental health peer support interventions to reduce the psychological treatment and support gap for patients who have been discharged from acute psychiatric care.

**Trial registration:**

clinicaltrials.gov, NCT05133726. Registered 24 November 2021

**Supplementary Information:**

The online version contains supplementary material available at 10.1186/s12913-022-07510-8.

## Background

Avoidable hospital readmission is a growing concern in health systems across the world. Readmissions often lead to significant physical, psychological, and financial impact on patients and their families. The health system is also affected as there are limited infrastructural, human, and financial resources [1, 2]. This is true in the era of the COVID-19 pandemic, which has seen many hospitals overwhelmed by rising emergency department (ED) presentations [3–5]. Consequently, there is a renewed interest to seek out solutions to mitigate avoidable readmissions, particularly in acute care.

People with psychiatric disorders have the highest early readmission rates among all hospitalized patients [6–8]. Early readmission is defined as readmission within 30 days of previous discharge [6]. Whereas deinstitutionalization of care and transition to community-based mental health care has been an approach of focus for decades [9–11], early hospital readmission rates remain high. Unmet need for psychological treatment and the limited human resources to address this gap is a major cause of high 30-day readmissions rates in acute psychiatry units [6].

In Alberta, Canada, about 8.4–11.9% of residents have a mental illness, but less than 25% of these individuals report that their mental health care needs were fully met [12]. This is despite the statistic that almost 70% of people with mental illnesses had accessed and utilized a provincial mental health service the year before, according to a 2014 mental health gap assessment report [12]. The greatest unmet need cited is the lack of sufficient, accessible, and affordable counselling. Their next greatest concern was an unmet need for tailored information on their own mental health challenges. This is despite several interventions provided by the provincial health authority, Alberta Health Services (AHS), to address addiction and mental health challenges through their community health clinics, and free-standing addiction and mental health (AMH) facilities, among others. Interestingly, 86% of AHS direct providers believe that they provide sufficient information to clients [12]. This discrepancy between service recipients and community providers’ perspectives reinforces concerns that services may not be well tailored to the diverse perceived needs of various groups. A consequence of this gap is the high demand for more specialist services and high readmissions after discharge from a recent hospital stay, leading to higher cost of health services [12, 13]. One study reports 90-day readmission rates of up to 14.0%, with the median time to readmission being 24 days [14]. More findings from the 2014 mental health gap assessment revealed that current management strategies are reactive, with system resources heavily invested in inpatient, residential and crisis services. Technology was noted to be under-utilized in the province. Reducing the felt need for hospital visits by providing alternative effective care will mitigate the existing strain on healthcare, human and financial resources due to mental illness [12, 13]. To address this, we propose an innovative program which augments mental health peer support, which is provided by individuals with lived experience of mental illness, with an evidence-based supportive text messaging program developed using the principles of cognitive behavioral therapy (CBT) [13].

### Components of the innovation

1. Peer support is valued in recovery-oriented models [15] of mental illness and increasingly implemented [16]. Evidence indicates positive effects, including lower inpatient service use, better relationships with providers and increased engagement. Peer Support Workers (PSWs) who are in recovery and have lived experience with mental illness will play central roles in this study, including delivering some of the interventions proposed. PSW activities will include supportive face-to-face visits, interactive phone calls/texts/zoom meetings with patients, advocacy, connecting patients with community resources, and experiential sharing. PSWs will be actively engaged through all provincial practice councils and are already embedded within many Alberta AMH programs. Evidence also shows that providing peer support to others may also benefit the PSWs by enhancing their own feelings of competence and personal value [17–22].
2. Text4Support will be provided through ResilienceNHope, which is an online application site (https://application.resiliencenhope.com/). It is a low-cost, evidence-based, supportive text messaging program which will be coupled with or without mental health peer support services to reduce the psychological treatment and support gap for study participants who have been discharged from acute psychiatry care into community. Starting a day after enrolment, intervention-group participants will receive daily unidirectional (no-reply) supportive text messages. The text messages will be written by cognitive behavior therapists in partnership with individuals with lived experience of mental illness. The Text4Support program was developed based on knowledge from randomized controlled trials conducted in Ireland [23, 24] and Alberta [13, 25]. The program is also based on knowledge gained from the highly successful award winning Text4Mood program in Alberta’s North Zone which improved the psychological treatment gap and was effective [26] and scalable (i.e., > 10,000 recipients within 6 months). The Text4Hope program was launched in Alberta during the COVID-19 pandemic which was also effective and scalable (> 48,000 subscribers within 3 months) [27–37]. Text4Support provides CBT-based and diagnosis-specific daily supportive text messages for 6 months. Some examples of the text messages are:

- What lies behind you and what lies before you are tiny matters compared to what lies within you. Have faith in yourself, and success can be yours.
- There are 2 days in the week we should not worry about, yesterday and tomorrow. That leaves today. Live for today.
- Stumbling blocks can become steppingstones to a better life. You can turn adversities into opportunities. Don’t be discouraged by today’s problems.
- Letting go of resentment is a gift you give yourself. It will ease your journey immeasurably. Make peace with everyone, and happiness will be yours.

Determination of diagnosis-specific approach was based on an analysis of Alberta service data, which suggested that diagnostic clusters for individuals discharged from acute psychiatry units into community for follow-up fell into six major categories: mood disorders, anxiety disorders, schizophrenia and other psychotic disorders, substance use disorders, adjustment disorders and personality disorders. Thus, text message content will focus on two dimensions. First, general content that is indicated regardless of symptomatology will be presented, including messages of self-care, social support, hope, affirmation, and recovery. Second, specific content will be provided that focuses on management of symptoms related to the specific conditions described above (e.g., activity scheduling in depression). As a component of scalability and inclusiveness, text message content can also be customized based on end-user characteristics. Specifically, we will explore content that is sensitive to needs based on age group, cultural identity, gender identity, and non-English language communication. This serves to enhance access for diverse groups, many of whom may be marginalized or underserved. In addition, individuals identified to be most at-risk of readmission to hospital will be offered mental health peer support. Matching an individual to a PSW will also consider the individual’s choice, demographic characteristics and factors, such as cultural and gender identity. These will be explored as the project is scaled up and the pool of PSWs increases.

### Study aim

This study is designed to address gaps in care/support available at the community level for individuals discharged from inpatient psychiatry units and referred to community mental health services for follow-up. We aim to reduce the psychological treatment and support gap for those who have been discharged from acute care and are scheduled to receive mental health and psychiatric treatment from AMH services after a long wait. Unpublished preliminary data from these pilot interventions provide evidence that psychiatric readmissions and emergency department visits are reduced by 10-25%. Given these results, if implemented at scale in Alberta could result in cost-savings for individuals and the province.

## Methods and analysis

This study will both evaluate the effectiveness of this intervention as well as implementation context and outcomes. Implementation context is “the set of circumstances or unique factors that surround a particular implementation effort” [38], while implementation outcomes are “effects of deliberate and purposive actions to implement new treatments, practices, and services” [39]. Implementation outcomes are indicators of implementation efforts, and are distinct from service or clinical outcomes [39].

The Consolidated Framework for Implementation Research (CFIR) will provide an overarching guidance to design, implementation, and evaluation by examining outer/inner contexts, intervention characteristics, and stakeholders involved as well as the process of implementation [40].

Further, using the Reach-Effectiveness-Adoption-Implementation- Maintenance (RE-AIM) framework [41], we will: (1) examine the reach of the interventions, i.e., text and peer support; (2) evaluate their effectiveness; (3) gauge support for their adoption; (4) evaluate fidelity in implementation; and (5) document the maintenance (sustainability) of implementation post-trail.

### Study design

**Fig. 1:**
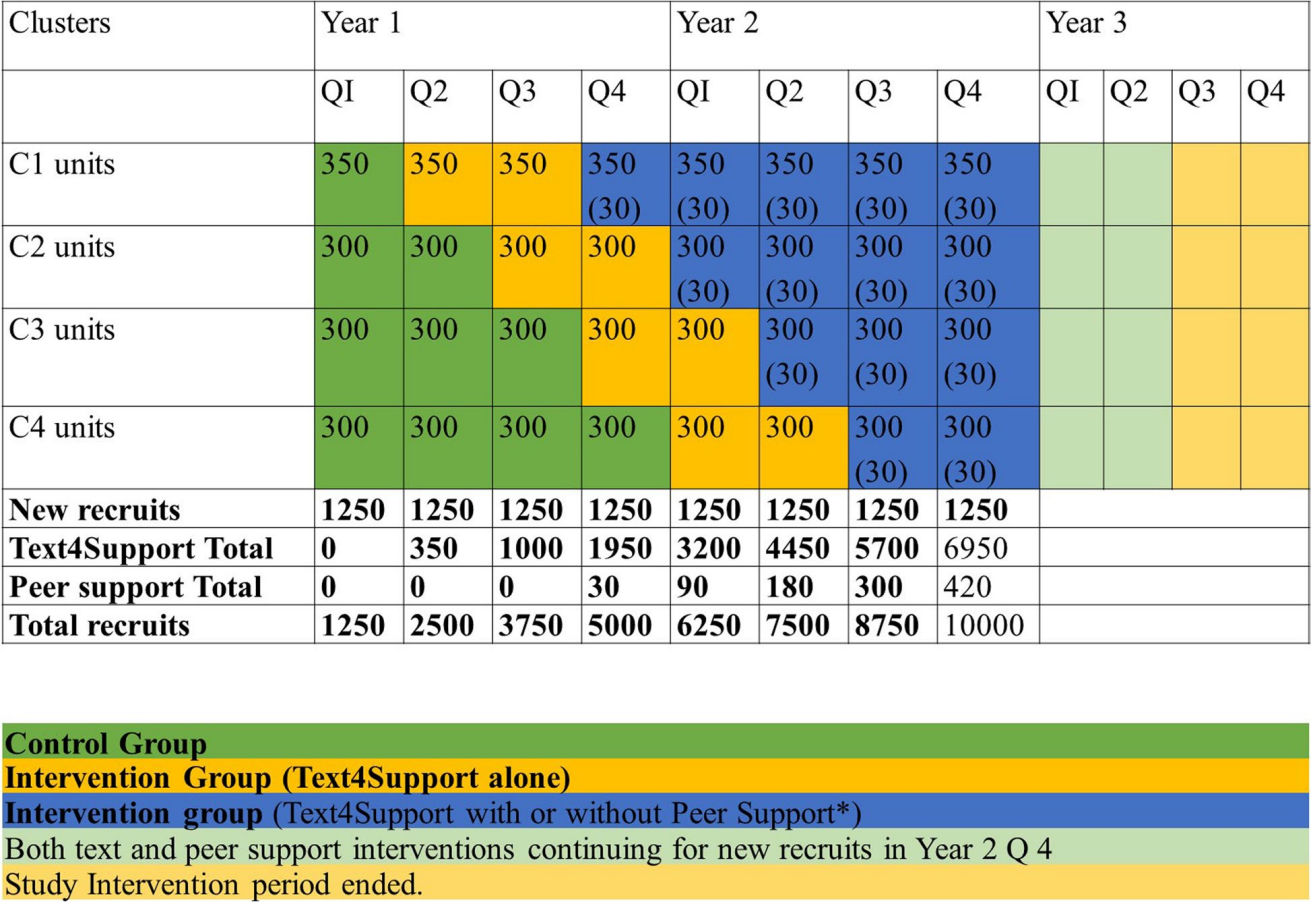
Number of new and total recruits expected in each cluster and period

A pragmatic stepped-wedge cluster-randomized approach will be applied, providing Text4Support and Peer Support Service (PSS) to 10,000 participants recruited across 9 acute care sites and day hospitals across Alberta as the clustered unit of randomization (Fig. 1). The design reconciles constraints under which policy makers and service managers operate with the need for rigorous scientific evaluations. In a stepped-wedge study, the design is extended so every cluster provides pre-post observations, and switches from control to intervention exposure but not at the same time-point [42].

The design is a preferable study design when:

- Evidence supports the intervention (e.g., known to be effective at individual level but uncertain about policy level), or there is resistance to parallel designs in which only half of the clusters receive the intervention.
- The intervention is a service delivery or policy change that can be implemented with the primary outcome, or at least some important outcomes available from routinely collected data (e.g., reduced readmission rates, acute care cost and no- show rates, routinely collected by AHS and primary study outcomes).
- The intra-cluster correlation is anticipated as high or cluster sizes large, so a cross-sectional stepped-wedge design is likely more efficient than a parallel cluster design.

This design has been successfully implemented with complex programs and change management involving large-scale programs in many countries [43, 44]. The study also adheres to checklists from the EQUATOR network ([45–47] (see Additional files 1 and 2).

### Participant recruitment

Individuals who have been diagnosed with a mental illness, are ready for discharge from an inpatient psychiatry unit that is part of the study, are 18 to 65 years of age, have a mobile device, can read English text messages, and can provide informed written consent will be eligible for the study. Recruitment will commence in February 2022 and will be done by the research team with physicians and other providers at the various sites. Participants should have a mobile device capable of receiving text messages. Participants will be ineligible if they know they will be based out of town during the 12-month follow-up period.

### Sample size considerations

We anticipate a reaching a target sample of 10,000. This figure is about 50% of expected psychiatric discharges, based on the 2018 administrative data (*n* = 20,747) [48]. We decided not to present detailed power analysis for sample size, which is controversial for stepped-wedge design approaches [49]. Further, our study is a pragmatic trial aiming to cause change in real-world conditions, rather than an explanatory trial which aims to study under ideal conditions. Thus, our approach is guided by the Pragmatic-Explanatory Continuum Indicator Summary 2 (PRECIS-2) toolkit with which we aim to align with the higher scores in pragmatism [50].

With our intended 5 randomized clusters and targeted overall project clinical population sample size of 10,000 (including accounting conservatively for 20% patient dropout and 10% dropout for PSWs), we are confident that our sample sizes are more than adequate as we expect at least medium-effect sizes, consistent with our prior results with supportive text messaging programs in Alberta [51].

### Outcome measures

This study will evaluate both effectiveness outcomes (measures of impact) and implementation outcomes (measures of process). The effectiveness outcomes are system utilization measures and patient outcome measures [52–54]. Implementation outcomes include the reach of the interventions, acceptability, appropriateness, fidelity, and cost-effectiveness (Table 1).

**Table 1:** Outcome measures

Outcome measure	Instrument	Description	Reference (instrument)
*Effectiveness outcomes*			
30-day and annual readmission rates and annual emergency services utilization	Administrative data	The number and proportion of patients readmitted into acute care units within 30-days and 365 days of last discharge.	-
Psychological wellbeing	The Clinical Outcomes in Routine Evaluation - 10 (CORE-10)	A 10- item instrument developed to monitor clinically significant change in outpatients. The CORE-10 covers four domains: Well-being, problems/symptoms, functioning, and risk, and sums up in two total scores: the mean of all items, and the mean of all non-risk items. These measures will be assessed at baseline, six and 12 months for each participant.	Skre et al. 2013 [52]
Quality of life	The 5-level EQ-5D version (EQ-5D-5L)	The EQ-5D-5L comprises of 2 sections: the EQ-5D descriptive system and the EQ visual analogue scale (EQ VAS). The descriptive system consists of five components: mobility, self-care, usual activities, pain/discomfort and anxiety/depression. Each component has 5 levels: no problems, slight problems, moderate problems, severe problems and extreme problems. The EQ VAS can be used as a quantitative measure of health outcome ranging from worst imaginable health to best imaginable health, reflecting the patient’s own judgement. These measures will be assessed at baseline, six and 12 months for each participant.	Herdman et al. 2011 [53]
Patient’s recovery	Recovery Assessment Scale (RAS)	The Recovery Assessment Scale (RAS) is a 20-item measure developed based on a process model of recovery. The RAS evaluates various aspects of recovery with a special focus on hope and self-determination. These measures will be assessed at baseline, six and 12 months for each participant	Giffort et al. 2000 [54]
Patient’s resilience	The Brief Resilience Scale	The Brief Resilience Scale evaluates the perceived ability to bounce back or recover from stress. These measures will be assessed at baseline, six and 12 months for each participant.	Smith et al. 2008 [55]
Client satisfaction/Experience surveys.	Instrument developed and pilot tested, and published by the authors	Evaluates clients’ satisfaction and experiences with supportive text messaging programs. These measures will be assessed at 12 months for each participant.	Shalaby et al. 2022 [56]
*Implementation outcomes*			
Reach	Administrative data	The proportion of target population who receive the daily supportive/reminder text message and peer support interventions across Alberta.	-
Acceptability	Instrument developed and pilot tested, and published by the authors	Evaluates clients’ satisfaction and experiences with supportive text messaging programs. These measures will be assessed at 12 months for each participant.	Shalaby et al. 2022 [56]
Appropriateness of the messages and peer support services	Sociocultural sensitivity, Gender sensitivity, Age sensitivity.	Qualitative in-depth interviews.	-
Fidelity	Administrative data	Percentage of scheduled peer support follow-up visits completed as planned. Adherence to the peer support guidelines These measures will be assessed at baseline, six and 12 months.	-
Incremental cost-utility of implementing both interventions	Administrative data	The incremental cost-effectiveness ratio (ICER) is the ratio between the difference in costs and the difference in benefits of two interventions. These measures will be assessed at baseline and 12 months.	

### Data analysis

We will conduct preliminary analysis at the end of Q2 (see Fig. 1) and continue to monitor clinical and economic results at each six-month period over years 1-3, culminating in final analysis at the end of Q2 year 3. Primary outcome measurements will be extracted from administrative data, and distribution of primary outcomes data across unexposed observation periods will be compared with the exposed observation periods. Characteristics of individuals/clusters will be summarized by exposure status to allow consideration of selection biases and lack of balance [42].

Using data from the first preliminary data analysis at the end of Q2 and refining this at each subsequent analysis, we will perform conventional regression methods to predict characteristics and risk factors for patients usually readmitted for inpatient psychiatric treatment within 30 days of discharge. We will further perform machine-learning analysis to predict individuals who may be at risk of readmission within 30 days of discharge at each step. After the first cluster is followed-up, at each step of the stepped-wedge process, we will train a machine-learning model based on existing clusters and test the accuracy of their predictions in the next cluster being followed-up. The potential predictors (features) will include all demographic variables (e.g., age, sex, gender, employment, relationships and housing status, education) and clinical/administrative variables (e.g., diagnosis, substance misuse co-diagnosis, mental and physical comorbidities, presentations to the ED or utilization of crisis services over the period, number of readmissions in the previous year, types of psychotropic medications patient is taking, depot injection or not, Community Treatment Order or not, whether the individual has a General Practitioner or not, and has significant family support or not). The candidate machine learning algorithms will include commonly-used regularized logistic regression based on ElasticNet [55], support vector machines (SVM) and random forest [56, 57]. We will perform feature-, model- and parameter-selection within the training data. The machine learning model could be used for future predictions of readmission but will only be validated in this project and will not be used to intervene the stepped-wedge approach, so that the internal validity and random sampling will be maintained. We hope by the end of the project, we will develop a machine learning model to differentially support individuals at risk of 30-day readmission within 30 days of their discharge with peer support.

Cost-utility evaluation will estimate expected incremental cost per Quality Adjusted Life Year (QALY) gained by implementing the peer support and Text4Support programs, within the Net Benefit Regression framework [58]. Differences in relevant participant cohort characteristics will be controlled for during analysis. A health system perspective and within-study analysis (only study cohort costs and outcomes) will be adopted in the evaluation, comparing resource use and health outcomes (health-related quality of life) for intervention and usual care cohorts. These will be combined to calculate within-study expected QALY for each cohort. The economic evaluation methods (e.g., discount rate choice and uncertainty characterization) will align with recent reference case recommendations [59].

To examine the mechanisms that relate the outcomes to the potential predictors, and to generate contextualized information about processes related to the proposed solution (i.e., the intervention), we will embed a one-phase qualitative component using qualitative descriptive methodology [60]. In this embedded experimental mixed methods design (also referred to as a concurrent nested model) [61], we will draw and invite a purposive sub-sample from the overall pool of study participants at the end of Q2 to participate in this study component. We will aim to recruit 25-30 participants. Data will be collected using semi-structured individual interviews conducted by trained research team members in person or via Zoom. The interview will last about 1 hour, and participants will be offered a $40 gift card stipend during the enrollment process to thank them for their time and involvement. The interviews will be audio-recorded, transcribed verbatim, and entered in NVIVO 12 software for data organization and preparation for analysis.

Qualitative descriptive (QD) methodology is an appropriate approach for qualitative research aimed at generating information to refine interventions in everyday terms [60]. Aligned with QD methods [60], qualitative content analysis (QCA) will be conducted to summarize the content of the data [62, 63].

## Discussion

Peer support [13] and text message interventions [14, 23] have advantageous impacts on readmission rates, acute care costs, and no-show rates. We expect our intervention to be equally or more effective than previous Text4Hope and Text4Mood interventions. This innovation mitigates geographical access barriers given that it is a text message-based intervention that can be accessed anywhere mobile network service is available.

The Text4Mood program in Alberta’s North Zone improved the psychological treatment gap [26]. With the Text4Mood program, 82% of respondents to a user survey felt the text messages made them more hopeful about managing issues in their lives. 77% thought the text messages made them feel in charge of managing depression and anxiety. 75% felt connected to a support system. Most respondents (83%) felt Text4Mood improved their overall mental well-being, thus impacting their need for hospital-based care. Similarly, the Text4Hope launched in Alberta in response to the COVID-19 pandemic recorded 32,805 subscriptions from Albertans within the first week [64]. This emergency intervention during the pandemic significantly mitigated the need for readmission, a risk for COVID-19 infection, by reducing mental health distress by over 20% during the pandemic [28]. A combination of this supportive messaging intervention with peer support may have an additive effect in improving outcomes. The use of digital health has become very imperative during the ongoing pandemic. The pragmatic nature of this innovation not only delivers evidence-based supportive text messages which can be deployed on any phone with texting function, irrespective of sophistication, it would also test the effectiveness and implementation of peer support delivered through e-platforms like zoom.

### Anticipated risks and limitations

Generally, this intervention portends little risk to participants, health workers, or the general public. Some participants may view this intervention as a replacement for outpatient or community face to face counseling post-discharge from acute care. However, this risk is neither supported by evidence from the literature or knowledge from implementing our predecessor program, the Text4Mood. Despite this, the PSS component of the Text4Support intervention will further eliminate the likelihood of this effect.

PSW burn-out is also a potential risk. To mitigate this risk, each PSW will be assigned a mental health therapist who will provide support and mentorship. Further, psychiatrists will be available to help where needed.

The ongoing COVID-19 pandemic and associated factors may pose risks to participants, researchers, and project implementation. To mitigate this, we will adhere strictly to the University of Alberta’s revised ethics guidelines for COVID-19 as well as the regularly updated guidelines and policies from the national and provincial authorities. Further, applying a pragmatic study design along with implementation science strategies and tools will help navigate the complex evolving study context caused by the pandemic’s other temporal trends. Operational level changes will be documented carefully, guided by RE-AIM and the Pragmatic-Explanatory Continuum Indicator Summary 2 (PRECIS-2) toolkit [50].

**Fig. 2:**
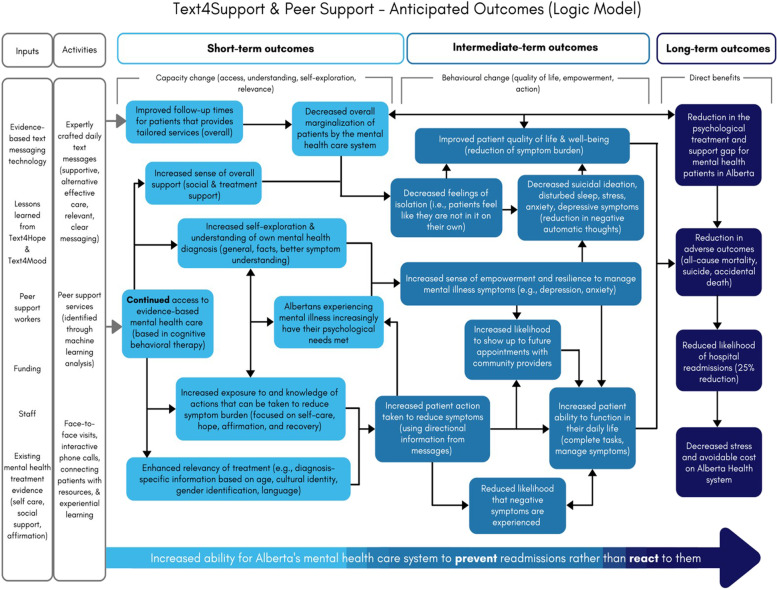
The Logic Model of the anticipated outcome for Text4Support & Peer Support program

We expect the results for the primary, secondary, and exploratory outcome measures to be available within 3 years of project commencement. It is expected that the results will shed further light on the feasibility of using supportive text message combined with peer support interventions in long-term care for discharged psychiatric inpatients in Alberta. The anticipated outcome of the Text4Support & Peer Support project is illustrated in the logic model attached (Details in Fig. 2).

## Conclusion

This study will demonstrate an innovative approach to providing community-based support to individuals with mental illnesses. The success of this intervention will not only provide evidence on a new management approach, but also contribute to knowledge on how to implement this innovation across varying contexts.

## Supplementary Information

**Additional file 1.** SPIRIT checklist

**Additional file 2.** TIDieR checklist

## Data Availability

Not applicable.

## Abbreviations

AMH: Addiction and mental health
AHS: Alberta Health Services
CORE-10: Clinical Outcomes in Routine Evaluation - 10
CBT: Cognitive behavioral therapy
CFIR: Consolidated Framework for Implementation Research
ED: Emergency department
HiMARC: Heroes in Mind, Advocacy and Research Consortium
PHQ-9: Patient Health Questionnaire
PSS: Peer Support Service
PSWs: Peer Support Workers
PRECIS-2: Pragmatic-Explanatory Continuum Indicator Summary 2
QCA: Qualitative content analysis
QD: Qualitative descriptive
QALY: Quality Adjusted Life Year
RE-AIM: Reach-Effectiveness-Adoption-Implementation- Maintenance
RAS: Recovery Assessment Scale
SVM: Support Vector Machines
